# ^68^Ga-PSMA-PET screening and transponder-guided salvage radiotherapy to the prostate bed alone for biochemical recurrence following prostatectomy: interim outcomes of a phase II trial

**DOI:** 10.1007/s00345-021-03735-0

**Published:** 2021-06-02

**Authors:** Patrick Bowden, Andrew W. See, Kevin So, Nathan Lawrentschuk, Daniel Moon, Declan G. Murphy, Ranjit Rao, Alan Crosthwaite, Dennis King, Hodo Haxhimolla, Jeremy Grummet, Paul Ruljancich, Dennis Gyomber, Adam Landau, Nicholas Campbell, Mark Frydenberg, Lloyd M. L. Smyth, Skye Nolan, Stella M. Gwini, Dean P. McKenzie

**Affiliations:** 1 Icon Cancer Centre, Richmond, VIC Australia; 2 Division of Cancer Surgery, Peter MacCallum Cancer Centre, Melbourne, VIC Australia; 3 Department of Urology, Royal Melbourne Hospital, Melbourne, VIC Australia; 4 Department of Surgery, University of Melbourne, Melbourne, VIC Australia; 5 EJ Whitten Centre for Prostate Cancer Research, Epworth Healthcare, Melbourne, VIC Australia; 6 Royal Melbourne Clinical School, University of Melbourne, Parkville, VIC Australia; 7 Sir Peter MacCallum Division of Oncology, University of Melbourne, Parkville, VIC Australia; 8 Epworth HealthCare, Richmond, VIC Australia; 9 Royal Melbourne Hospital, Parkville, VIC Australia; 10 Epworth Eastern, Box Hill, VIC Australia; 11 Royal Australian College of Surgeons, East Melbourne, VIC Australia; 12 Cabrini Hospital, Malvern, VIC Australia; 13 Department of Urology, The Canberra Hospital, Garran, ACT Australia; 14 Department of Surgery, Central Clinical School, Monash University, Melbourne, VIC Australia; 15 Department of Surgery, University of Melbourne, Heidelberg, VIC Australia; 16 Urology Department, Eastern Health, Box Hill, VIC Australia; 17 Bairnsdale Regional Health Service, Bairnsdale, VIC Australia; 18 Department of Surgery, Monash University, Clayton, VIC Australia; 19 Australian Urology Associates, Melbourne, VIC Australia; 20 School of Public Health and Preventive Medicine, Monash University, Clayton, VIC Australia

**Keywords:** Biochemical recurrence, Imaging, Positron emission tomography, Prostate cancer, Prostate-specific membrane antigen, Radiotherapy

## Abstract

**Purpose:**

To evaluate outcomes for men with biochemically recurrent prostate cancer who were selected for transponder-guided salvage radiotherapy (SRT) to the prostate bed alone by ^68^Ga-labelled prostate-specific membrane antigen positron emission tomography (^68^Ga-PSMA-PET).

**Methods:**

This is a single-arm, prospective study of men with a prostate-specific antigen (PSA) level rising to 0.1–2.5 ng/mL following radical prostatectomy. Patients were staged with ^68^Ga-PSMA-PET and those with a negative finding, or a positive finding localised to the prostate bed, continued to SRT only to the prostate bed alone with real-time target-tracking using electromagnetic transponders. The primary endpoint was freedom from biochemical relapse (FFBR, PSA > 0.2 ng/mL from the post-radiotherapy nadir). Secondary endpoints were time to biochemical relapse, toxicity and patient-reported quality of life (QoL).

**Results:**

Ninety-two patients (median PSA of 0.18 ng/ml, IQR 0.12–0.36), were screened with ^68^Ga-PSMA-PET and metastatic disease was found in 20 (21.7%) patients. Sixty-nine of 72 non-metastatic patients elected to proceed with SRT. At the interim (3-year) analysis, 32 (46.4%) patients (95% CI 34.3–58.8%) were FFBR. The median time to biochemical relapse was 16.1 months. The rate of FFBR was 82.4% for ISUP grade-group 2 patients. Rates of grade 2 or higher gastrointestinal and genitourinary toxicity were 0% and 15.2%, respectively. General health and disease-specific QoL remained stable.

**Conclusion:**

Pre-SRT ^68^Ga-PSMA-PET scans detect metastatic disease in a proportion of patients at low PSA levels but fail to improve FFBR. Transponder-guided SRT to the prostate bed alone is associated with a favourable toxicity profile and preserved QoL.

**Trial registration number:**

ACTRN12615001183572, 03/11/2015, retrospectively registered.

**Supplementary Information:**

The online version contains supplementary material available at 10.1007/s00345-021-03735-0.

## Introduction

One in three men with prostate cancer (PCa) will experience recurrence within 10 years of radical prostatectomy (RP), initially presenting as a rising serum prostate-specific antigen (PSA) level [1]. The recommended treatment for these patients is salvage radiotherapy (SRT) to the prostate bed at the earliest sign of biochemical recurrence [2], ideally when PSA levels are less than 0.5 ng/mL [3].

However, conventional imaging modalities have poor sensitivity to metastatic disease at these PSA levels [3]. Therefore, clinicians must estimate the likelihood that a patient has locally recurrent, non-metastatic disease to reduce the risk of futile local salvage therapy. Elective pelvic nodal irradiation and/or androgen deprivation therapy (ADT) can be added to SRT for patients at higher risk of microscopic metastatic disease. While ADT may improve progression-free [4] and overall [5] survival, these benefits come at the expense of reduced quality of life (QoL) and increased mortality from other causes [5–8]. Therefore, men with recurrence locally in the prostate bed alone are at risk of overtreatment.

^68^Ga-labelled prostate-specific membrane antigen positron emission tomography (^68^Ga-PSMA-PET) is superior to conventional imaging for staging patients with newly diagnosed high-risk PCa [9], providing improved imaging accuracy and sensitivity for men who are candidates for salvage therapy [10, 11]. Compared to the findings of conventional imaging, ^68^Ga-PSMA-PET can change the treatment strategy in up to two-thirds of patients [12], leading to reduced use of ADT and the increased deployment of PET-directed local therapy [13].

Real-time target-tracking via implantable electromagnetic transponders is a further technological advancement which can optimise the delivery of SRT in the post-prostatectomy setting [14], however, long-term oncological and toxicity outcomes have not been previously reported. Movement of the prostate bed during SRT is not trivial, as it increases the risk of rectal toxicity and undertreatment of the target [15].

The purpose of this study was to prospectively evaluate clinical outcomes over 10 years for patients offered transponder-guided SRT to the prostate bed alone, without ADT, based on a negative ^68^Ga-PSMA-PET result or a positive finding in the prostate bed only. Herein are the interim (3-year) results, describing the rate of freedom from biochemical relapse (FFBR), toxicity and patient-reported health-related QoL.

## Patients and methods

### Study design, setting and patients

This was a prospective, single-centre, single-arm cohort study of men with biochemically recurrent PCa following RP. The study was approved by the Epworth Human Research Ethics Committee (675-15). All participants provided informed consent.

Eligible patients had previously undergone a RP for histologically confirmed, clinical stage T1–3 PCa, and subsequently presented with a rising PSA in the range of 0.1–2.5 ng/mL. Patients with metastatic disease outside the prostate bed, significant sarcomatoid, ductal or spindle-cell histology or ADT within 6 months prior to enrolment were excluded.

### Outcomes

The primary endpoint was the rate of FFBR following SRT. Biochemical relapse was defined as the first increase in PSA greater than 0.2 ng/mL from the post-radiotherapy nadir, given that there was a second confirmatory reading [3]. Secondary endpoints were: (i) time from the end of SRT to biochemical relapse, (ii) acute (≤ 6 weeks) and late toxicity (> 6 weeks), defined by the Common Terminology Criteria for Adverse Events (version 4.03) and, (iii) changes in patient-reported general health and disease-specific QoL from baseline, assessed via the Short Form 12 Health Survey (SF-12, version 1) and Expanded Prostate Cancer Index Composite Short Form (EPIC-26), respectively.

### Trial procedures

The study was conducted in two phases. First, eligible patients were screened with ^68^Ga-PSMA-PET, in addition to a standard-of-care contrast-enhanced CT scan of the chest, abdomen and pelvis. A low-dose whole-body CT scan was performed simultaneously to the PET scan and the imaging datasets fused to assist with image interpretation. All images were reviewed by an experienced nuclear medicine physician and equivocal findings were discussed by a multidisciplinary team.

Patients were included in the subsequent treatment phase of the trial if they had a negative ^68^Ga-PSMA-PET result or a positive finding limited to the prostate bed only. Eligible patients received SRT to the prostate bed alone to a total dose of 70 Gray (Gy) in 35 fractions (^68^Ga-PSMA-PET negative) or 74Gy in 37 fractions (^68^Ga-PSMA-PET positive in the prostate bed) [16]. Patients with a suitable body habitus (anterior–posterior separation between the anterior skin surface and greater trochanter notch less than 17 cm when lying supine) were implanted with three electromagnetic transponders in the prostate bed for real-time target-tracking using the Calypso^®^ localisation system (Varian Medical Systems, Palo Alto, CA) during treatment. Transponders were implanted via a transperineal route under ultrasound guidance. Contraindications for transponder implantation were the presence of metal prosthetic implants in the pelvis and the use of anti-coagulant or anti-platelet therapy.

The clinical target volume (CTV) included the entire prostate bed, and for patients with relevant pathological features, the region of the seminal vesicle bed at risk of microscopic disease [17]. Magnetic resonance imaging was used to assist with target volume delineation when the planning CT scan was adversely affected by image artefact. For patients with Calypso^®^ transponders, the planning target volume (PTV) was an isotropic 5-mm expansion of the CTV. For patients without transponders, the expansion was 5 mm posteriorly and 10 mm in all other directions from the CTV.

Treatment was delivered using intensity-modulated radiotherapy with daily corrections for isocentre position via dual orthogonal kV imaging and a weekly (at minimum) cone-beam CT scan. For patients with transponders, the Calypso^®^ system gated treatment when transponders indicated that the target was greater than 3 mm from the planned position.

### Follow-up

Follow-up was conducted 6 weeks after SRT, quarterly for the next 2 years, then half-yearly thereafter, until a patient reached the primary endpoint of biochemical relapse. Each follow-up point included PSA testing, clinical evaluation, toxicity assessment and administration of QoL questionnaires.

### Statistical analysis

Data were analysed using the Stata statistical package (version 16, Stata Corporation, College Station, TX). Categorical data were summarised using frequencies and percentages while continuous/interval data were shown as means (with standard deviation) or medians with the interquartile range (IQR). Chi-squared test, *t* test and Wilcoxon rank-sum test were used to compare characteristics of non-metastatic and metastatic patients.

FFBR was reported as a percentage with the 95% confidence interval (CI). The relationship between biochemical relapse and patients’ clinical characteristics was examined using logistic regression, with results reported as odds ratios (OR) with 95% CI. Additionally, Cox proportional hazards model was used to assess if time to relapse varied by patient characteristics, with findings reported as hazard ratio (HR) and 95% CI. A proportional hazards test based on Schoenfeld residuals was also conducted to ensure the proportional hazards assumption was satisfied [18].

Change in QoL over time was examined using random effects models and results were reported as difference from baseline with the 95% CI. QoL data were also evaluated by a minimal clinically important difference (MCID) in scores compared to baseline. The MCID was set to one-third of a standard deviation at baseline for each specific sub-domain [19, 20].

A conservative critical alpha value of *P* < 0.01 was employed for both the primary and secondary endpoints.

## Results

### Participants

Ninety-two men were enrolled into the screening phase of the trial between July 2015 and January 2017. In 20 (21.7%) patients, there was a positive ^68^Ga-PSMA-PET/CT finding beyond the prostate bed. Baseline characteristics are presented in Table 1 and split into two groups: non-metastatic (negative ^68^Ga-PSMA-PET or positive ^68^Ga-PSMA-PET finding in the prostate bed only) and metastatic (a positive ^68^Ga-PSMA-PET finding outside the prostate bed). The majority (75%) of patients with non-metastatic PCa had received RP, while 13 of 20 (65%) of patients in the metastatic group had RP with lymph-node dissection (*P* = 0.001), however, this difference was attenuated by adjusting for ISUP grade-group, pathological T stage and pre-surgery PSA (adjusted *P* = 0.07).

**Table 1:** Baseline characteristics of metastatic and non-metastatic patients as defined by ^68^Ga-PSMA-PET imaging in the screening phase of the trial

Characteristic	Metastatic (*n* = 20)	Non-Metastatic (*n* = 72)	Comparison of the two groups (*P *value)
Age mean (SD)	64.1 (7.0)	63.2 (6.5)	0.598
Pre-surgery PSA (ng/mL) median [IQR]	6.75 [4.70, 9.70]	7.90 [6.25, 12.80]	0.117
Missing *n* (%)	4 (30.8)	9 (69.2)	
Surgery type *n* (%)			
Radical prostatectomy	7 (11.5)	54 (88.5)	0.001
Radical prostatectomy and LN dissection	13 (31.9)	18 (68.1)	
ISUP grade-group *n* (%)			
1	0 (0)	2 (100.0)	0.452
2	3 (15.0)	17 (85.0)	
3	8 (19.0)	34 (81.0)	
4–5	9 (32.1)	19 (67.9)	
Pathological T stage *n* (%)			
T2 (any)	7 (29.2)	17 (70.8)	0.015
T3a	4 (9.1)	40 (90.9)	
T3b	7 (43.8)	9 (56.2)	
Missing	2 (25.0)	6 (75.0)	
Perineural invasion *n* (%)			
Absent	3 (25.0)	9 (75.0)	0.832
Present	17 (21.8)	61 (78.2)	
Missing	0 (0)	2 (100.0)	
Lymphovascular invasion *n* (%)			
Absent	12 (17.6)	56 (82.4)	0.136
Present	8 (36.4)	14 (63.6)	
Missing	0 (0)	2 (100.0)	
Pathological margin status *n* (%)			
Negative	14 (25.0)	42 (75.0)	0.606
Positive	6 (18.7)	26 (81.3)	
Missing	0 (0)	4 (100.0)	
Post-surgery PSA *n* (%)			
Undetectable	3 (9.1)	30 (90.9)	0.003
Persistent (≥ 0.1 ng/mL)	3 (20.0)	12 (80.0)	0.739
Pre-PSMA PSA (ng/mL) median [IQR]	0.37 [0.15, 0.84]	0.17 [0.12, 0.26]	0.002
Months between surgery and PSMA median [IQR]	13.0 [6.2, 47.8]	26.0 [12.0, 56.7]	0.154

*IQR* Interquartile range; *LN* lymph node; *PSA* prostate-specific antigen; *PSMA* prostate-specific membrane antigen; *SD* standard deviation

Three patients in the non-metastatic group withdrew from the trial following screening, leaving a total of 69 men to proceeded to the treatment phase of the trial. Four patients had a local recurrence in the prostate bed. Treatment was delivered with real-time target-tracking for 61 (88.4%) patients. The median follow-up following SRT was 34.7 months (IQR 13.1–49.0 months).

### Biochemical relapse

At the time of interim analysis, 32 (46.4%) patients (95% CI 34.3–58.8%) were FFBR, having a median follow-up of 49.0 months (IQR 43.6–49.9). Seventeen (24.6%) of these patients had an undetectable PSA reading. Of the 37 patients who failed biochemically, 21 had no radiographic evidence of metastatic disease, 3 had out-of-field seminal vesicle bed recurrence, 9 had locoregional nodal or bony metastases and 4 had distant metastases. Risk of relapse was significantly higher for ISUP grade groups 4–5 and 3 compared to grade group 2 (Table 2). No other variable was associated with relapse at *P* < 0.01.

**Table 2:** Relationship between biochemical relapse following salvage radiotherapy and patient characteristics

Characteristic	Biochemical relapse: *n* (%)		OR (unadjusted)	95% CI	*P* value
	No (*n* = 32)	Yes (*n* = 37)			
Surgery type					
Radical prostatectomy	24 (47.1)	27 (52.9)	Ref		
Radical prostatectomy and lymph-node dissection	8 (44.4)	10 (55.6)	1.11	0.37–3.30	0.849
ISUP grade-group					
2	14 (82.4)	3 (17.7)	Ref		
3	12 (37.5)	20 (62.5)	7.78	1.83–33.11	0.006
4–5	5 (26.3)	14 (73.7)	13.07	2.58–66.27	0.002
Pathological T stage					
T2 (any)	7 (43.8)	9 (56.3)	Ref		
T3a	21 (52.5)	19 (47.5)	0.70	0.22–2.28	0.558
T3b	0 (0)	7 (100.0)	–		
Surgical margins					
Negative	18 (42.9)	24 (57.1)	Ref		
Positive	13 (50.0)	13 (50.0)	0.75	0.28–2.02	0.569
Perineural invasion at surgery					
Absent	6 (75.0)	2 (25.0)	Ref		
Present	26 (44.1)	33 (55.9)	3.81	0.70–20.71	0.122
Lymphovascular invasion at surgery					
Absent	30 (55.6)	24 (44.4)	Ref		
Present	2 (15.4)	11 (84.6)	6.88	1.37–34.44	0.019
Post-surgical PSA undetectable					
Yes	14 (46.7)	16 (53.3)	Ref		
No	15 (41.7)	21 (58.3)	1.23	0.46–3.28	0.686
PSA Persistence (Post-surgical PSA ≥ 0.1 ng/mL)					
No	25 (45.5)	30 (54.5)	Ref		
Yes	4 (36.4)	7 (63.6)	1.46	0.38–5.62	0.583
Pre-PSMA PSA (ng/mL)	0.17 [0.12, 0.29]	0.15 [0.12, 0.26]	2.11	0.45–9.78	0.341
Pre-PSMA PSA ≥ 0.2 ng/mL					
No	21 (48.8)	22 (51.2)	Ref		
Yes	10 (40.0)	15 (60.0)	1.43	0.52–3.92	0.484
Months between surgery and PSMA median [IQR]	31.2 [20.1, 78.7]	22.4 [10.3, 34.2]	0.98	0.97–1.00	0.010

*CI* confidence interval; *OR* odds ratio; *PSA* prostate-specific antigen; *PSMA* prostate-specific membrane antigen; *Ref *reference group

The median time to biochemical relapse was 16.1 (IQR 6.9–25.5) months. Factors associated with a shorter time to relapse (Supplementary Table 1) were ISUP grade group 4–5 (HR 7.90, 95% CI 2.27–27.49, *P* = 0.001) or 3 (HR 5.44, 95% 1.66–17.80, *P* = 0.005), the presence of lymphovascular invasion (HR 2.34, 95% CI 1.23–4.44, *P* = 0.009) and pre-PSMA PSA (HR 2.65, 95% CI 1.35–5.18, *P* = 0.004).

### Toxicity

Acute toxicity was assessed in 62 patients. The maximum acute genitourinary (GU) toxicity was grade 1 or grade 2 for 13 (21.0%) patients and 1 (1.6%) patient, respectively, with urinary incontinence being reported most frequently (*n* = 8). Seven (11.3%) patients had a maximum grade 1 GI toxicity, most commonly proctitis (*n* = 4). No grade 2 or higher acute GI toxicities were reported.

Sixty-six patients had late toxicity assessments. Three patients experienced biochemical progression prior to having a late toxicity assessment. There was one (1.5%) late grade 3 adverse event (urethral stricture) attributable to SRT. The maximum late GU toxicity was grade 1 and grade 2 in 30 (45.5%) and 10 (15.2%) patients, respectively, with urinary incontinence being reported most frequently (grade 1, *n* = 23; grade 2, *n* = 9). Twelve (18.2%) patients had a maximum grade 1 late GI toxicity. No grade 2 or higher late GI toxicities were recorded.

### Quality of life

There were no clinically or statistically significant differences in physical component summary or mental component summary score from baseline up to 3 years post-treatment (Supplementary Fig. 1A). Disease-specific QoL for the cohort also remained within the MCID thresholds across all four sub-domains (Supplementary Fig. 1B).

## Discussion

To our knowledge, this is the first prospective study of ^68^Ga-PSMA-PET-guided SRT to the prostate bed alone, without ADT, for men with biochemically recurrent PCa following RP. At the interim analysis, 46.4% of patients were FFBR. There were no grade 2 or higher GI toxicities and one treatment-related grade 3 urinary toxicity after a median follow-up of 34.7 months. QoL was preserved across all general health and disease-specific sub-domains. Risk factors for biochemical relapse or time to biochemical relapse were ISUP grade-group ≥ 3 and lymphovascular invasion, consistent with previous studies [21–23].

SRT to the prostate bed alone, with no ADT, has historically been associated with 3–5-year rates of biochemical progression-free survival (bPFS) in the order of 50% [24, 25]. The 5-year interim results of the recent NRG Oncology/RTOG 0534 SPPORT trial report higher rates of bPFS (71%), however, the threshold for biochemical failure was 2 ng/mL above the post-SRT [26] nadir compared to 0.2 ng/mL in the present study. In addition, over 50% of patients had pathological T2 stage disease [26] (versus 23% in the present study).

The lack of improvement in the rate of FFBR in the present study compared to historical data is likely to reflect the limitations of ^68^Ga-PSMA-PET in the early SRT setting. A meta-analysis of 14 studies using ^68^Ga-PSMA-PET re-staging for biochemical recurrence after prostatectomy found that the rate of positive scans was 46% and 33% when the pre-imaging PSA was 0.2–0.49 ng/mL and 0–0.19 ng/mL, respectively [27]. The median pre-PSMA PSA level of non-metastatic patients in this study was 0.17 ng/mL (IQR 0.12–0.26), making it likely that a proportion of patients had microscopic disease outside the prostate bed despite a negative PSMA-PET finding. Therefore, early salvage treatment strategies should still be guided primarily by known risk factors for metastatic disease and options including ADT and pelvic node irradiation should be considered by both the patient and clinician.

Contemporary studies of SRT which add ADT and/or extended pelvic radiotherapy report rates of 3–5-year bPFS ranging from 47 to 83% [8, 21, 26, 28]. In a study also utilising pre-SRT ^68^Ga-PSMA-PET screening, Emmett et al. [28], report 81% FFBR at 3 years for patients with negative or prostate bed confined disease on imaging, similarly using a threshold for biochemical failure of 0.2 ng/mL above the post-SRT nadir. However, nearly half of these patients also had radiotherapy to their pelvic nodes and 14% received ADT. The rate of FFBR for patients with ISUP grade-group 2 disease in the present study was 82.4% at a median follow-up of over 4 years, suggesting the possibility of long-term biochemical control outcomes equivalent to those treated more aggressively with ADT and/or elective pelvic node irradiation. In contrast, 73.4% of patients with ISUP grade-group 4–5 disease relapsed, suggesting an upfront role for ADT and pelvic node irradiation in the salvage setting, even with a negative ^68^Ga-PSMA-PET scan.

The potential extension of progression-free survival associated with ADT [4, 5], however, must be considered in the context of substantially more toxicity in sexual and hormonal domains which can outlast the duration of treatment by up to a year, while testosterone levels normalise [29]. Studies adding ADT to SRT report substantially increased rates of grade 2 or higher hot flushes, sweating and gynaecomastia [5, 8]. In the present study, sexual and hormonal QoL were preserved up to 3 years post-treatment. The trade-off between disease control and QoL detriment contributes to a lack of consensus regarding the use of ADT at the time of SRT [3, 30].

Finally, the rates of toxicity in this study compare favourably to contemporary trials using SRT alone. Rates of late grade 2 or higher GU and GI toxicity are reported to be 31–38% and 10–15%, respectively [5, 26] and grade 3 or worse GU toxicity is reported in up to 8% of patients [5, 8, 26]. The lower rates of late grade 2 or higher GU (17%) and GI (0%) toxicity in the present study are likely to reflect the use of intensity-modulated, transponder-guided radiotherapy with real-time target tracking.

This study has a number of limitations. Several of the sub-groups are small and may have limited power to detect significant differences in FFBR. The number of patients with a positive ^68^Ga-PSMA-PET in the prostate bed only was too small for a sub-group analysis. Second, there was no comparator arm in this study, preventing an assessment of outcomes following SRT alone against other treatment strategies. Finally, this interim analysis presents toxicity outcomes at a median follow-up of 34.7 months, which is too early to evaluate the true rate of late urinary sequelae. The final results of this study will be reported at 10 years post-treatment.

In conclusion, the interim results of this study show that ^68^Ga-PSMA-PET screening prior to SRT to the prostate bed alone fails to detect metastatic disease in the majority of patients who subsequently relapse. These findings are indicative of the limitations of ^68^Ga-PSMA-PET in the early salvage setting. However, there is a subset of patients (ISUP grade-group 2) with a rising PSA post-prostatectomy who are at a low risk of metastases and can avoid overtreatment while expecting durable bPFS and preserved QoL using Calypso^®^ transponder-guided SRT.

## Supplementary Information

Below is the link to the electronic supplementary material.

Supplementary Table 1. Relationship between time to biochemical relapse and patient characteristics. (PDF 181 KB)

Supplementary Figure 1. Mean scores for general health (A) and disease specific (B) sub-domains. Quality of life remained stable over the course of three years for both general health (A) and disease specific (B) subdomains, as measured by SF-12 and EPIC-26 patient-reported quality of life instruments. Error bars reflect one standard deviation. Mean scores remained within minimal clinically important difference thresholds, signified by the pink shaded regions (+/− one third of a standard deviation of baseline scores, per sub-domain). In addition, there were no statistically significant differences in score compared to baseline on regression analysis. (PDF 48 KB)

## Data Availability

The datasets analysed during the current study are available from the corresponding author on reasonable request.
