# Supplementary material for: 68Ga-PSMA-PET screening and transponder-guided salvage radiotherapy to the prostate bed alone for biochemical recurrence following prostatectomy: interim outcomes of a phase II trial
Source: World J Urol. 2021 Jun 2;39(11):4117–25. doi: 10.1007/s00345-021-03735-0 (PMC8571130; doi:10.1007/s00345-021-03735-0)
Supplement: Supplementary file 1 — Supplementary Table 1. Relationship between time to biochemical relapse and patient characteristics. (PDF 181 KB) [file 345_2021_3735_MOESM1_ESM.pdf]

Title: ^{68}Ga-PSMA-PET screening and transponder-guided salvage radiotherapy to the prostate bed alone for biochemical recurrence following prostatectomy: interim outcomes of a phase II trial.

Authors: Patrick Bowden, Andrew W. See, Kevin So, Nathan Lawrentschuk, Daniel Moon, Declan G. Murphy, Ranjit Rao, Alan Crosthwaite, Dennis King, Hodo Haxhimolla, Jeremy Grummet, Paul Ruljancich, Dennis Gyomber, Adam Landau, Nicholas Campbell, Mark Frydenberg, Lloyd M. L. Smyth, Skye Nolan, Stella M. Gwini, Dean P. McKenzie

Corresponding author: Dr Patrick Bowden, Icon Cancer Centre, Richmond, VIC, Australia, email: pat.bowden@icon.team

Supplementary Figure 1

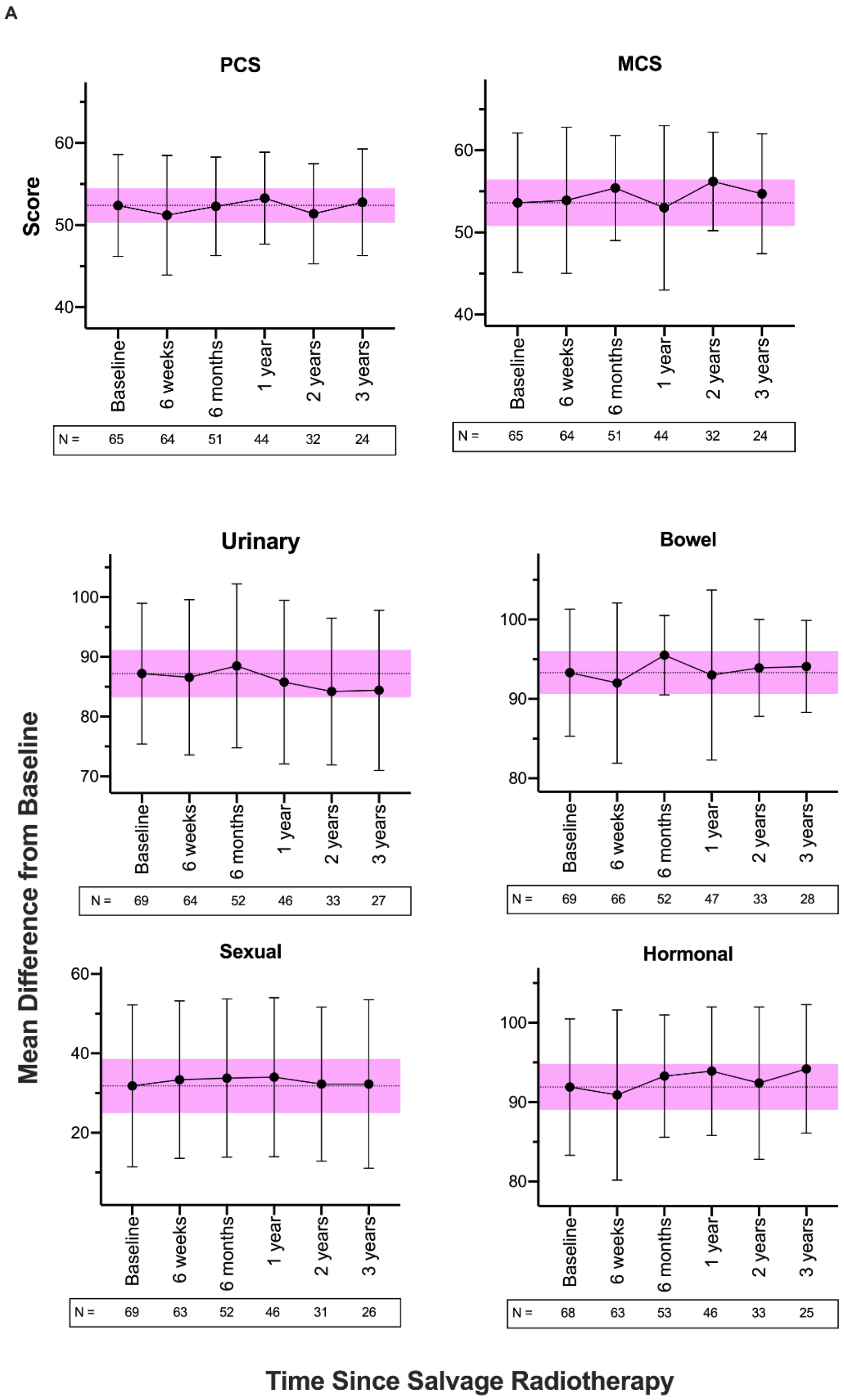

Supplementary Fig. 1 Mean scores for general health (A) and disease specific (B) sub-domains remained stable over the course of three years, as measured by SF-12 and EPIC-26 patient-reported quality of life instruments. Error bars reflect one standard deviation. Mean scores remained within minimal clinically important difference thresholds, signified by the pink shaded regions (+/- one third of a standard deviation of baseline scores, per sub-domain). In addition, there were no statistically significant differences in score compared to baseline on regression analysis
