# Supplementary material for: 68Ga-PSMA-PET screening and transponder-guided salvage radiotherapy to the prostate bed alone for biochemical recurrence following prostatectomy: interim outcomes of a phase II trial
Source: World J Urol. 2021 Jun 2;39(11):4117–25. doi: 10.1007/s00345-021-03735-0 (PMC8571130; doi:10.1007/s00345-021-03735-0)
Supplement: Supplementary file 2 — Supplementary Figure 1. Mean scores for general health (A) and disease specific (B) sub-domains. Quality of life remained stable over the course of three years for both general health (A) and disease specific (B) subdomains, as measured by SF-12 and EPIC-26 patient-reported quality of life instruments. Error bars reflect one standard deviation. Mean scores remained within minimal clinically important difference thresholds, signified by the pink shaded regions (+/− one third of a standard deviation of baseline scores, per sub-domain). In addition, there were no statistically significant differences in score compared to baseline on regression analysis. (PDF 48 KB) [file 345_2021_3735_MOESM2_ESM.pdf]

Title: ^{68}Ga-PSMA-PET screening and transponder-guided salvage radiotherapy to the prostate bed alone for biochemical recurrence following prostatectomy: interim outcomes of a phase II trial.

Authors: Patrick Bowden, Andrew W. See, Kevin So, Nathan Lawrentschuk, Daniel Moon, Declan G. Murphy, Ranjit Rao, Alan Crosthwaite, Dennis King, Hodo Haxhimolla, Jeremy Grummet, Paul Ruljancich, Dennis Gyomber, Adam Landau, Nicholas Campbell, Mark Frydenberg, Lloyd M. L. Smyth, Skye Nolan, Stella M. Gwini, Dean P. McKenzie

Corresponding author: Dr Patrick Bowden, Icon Cancer Centre, Richmond, VIC, Australia, email: pat.bowden@icon.team

Supplementary Table 1. Relationship between time to biochemical relapse and patient characteristics

Characteristic	HR	95% CI	p-value
Surgery Type			
Radical prostatectomy	Ref.		
Radical prostatectomy & LN dissection	1.22	0.57 – 2.63	0.611
ISUP grade-group			
2	Ref.		
3	5.44	1.66 – 17.80	*0.005*
4-5	7.90	2.27 – 27.49	*0.001*
Pathological T Stage			
T2 (any)	Ref.		
T3a	0.99	0.46 – 2.12	0.971
T3b	2.54	1.22 – 5.26	0.013
Surgical Margins			
Negative	Ref.		
Positive	0.85	0.43 – 1.69	0.651
Perineural invasion at surgery			
Absent	Ref.		
Present	3.02	0.81 – 11.18	0.099
Lymphovascular invasion at surgery			
Absent	Ref.		
Present	2.34	1.23 – 4.44	*0.009*
Post-surgical PSA undetectable			
Yes	Ref.		
No	1.22	0.64 – 2.31	0.548
PSA Persistence (Post-surgical PSA ≥ 0.1 ng/mL)			
No	Ref.		
Yes	1.90	0.75 – 4.81	0.173
Pre-PSMA PSA (ng/mL)	1.52	0.77 – 3.01	0.227
Months between surgery and PSMA	0.99	0.97 – 1.00	0.023

Abbreviations: CI, confidence interval; LN, lymph node; PSA, prostate-specific antigen; PSMA, prostate-specific membrane antigen; HR, hazard ratio; Ref., reference group
